# *Sekentei* and objectively-measured physical activity among older Japanese people: a cross-sectional analysis from the NEIGE study

**DOI:** 10.1186/s12889-019-7702-4

**Published:** 2019-10-22

**Authors:** Hiroshi Murayama, Shiho Amagasa, Shigeru Inoue, Takeo Fujiwara, Yugo Shobugawa

**Affiliations:** 1 Institute of Gerontology, The University of Tokyo, 7-3-1 Hongo, Bunkyo-ku, Tokyo, 113–8656 Japan; 2 Department of Preventive Medicine and Public Health, Tokyo Medical University, 6-1-1 Shinjuku, Shinjuku-ku, Tokyo, 160-8402 Japan; 3 Department of Global Health Promotion, Tokyo Medical and Dental University, 1-5-45, Yushima, Bunkyo-ku, Tokyo, 113-8510 Japan; 4 Division of International Medicine, Niigata University Graduate School of Medical and Dental Sciences, 1-757, Asahimachi-dori, Niigata City, Niigata 951-8510 Japan

**Keywords:** Japan, Older people, Physical activity, *Sekentei*, Social appearance, Social norm

## Abstract

**Background:**

The concept of *sekentei* (social appearance), defined as sensitivity about one’s reputation, reflects Japanese behavioral principles and involves pressure to conform to social norms, particularly among people living in rural areas. However, data regarding the relationship between *sekentei* and health behaviors are sparse. In this study, we examined the relationship between *sekentei* and objectively-measured physical activity among community-dwelling older people in Japan.

**Methods:**

We used data from the Neuron to Environmental Impact across Generations Study (NEIGE Study), which is a prospective cohort study of randomly-sampled community-dwelling individuals aged 65–84 years living in Tokamachi City, Niigata Prefecture, Japan. The baseline survey was conducted in 2017 and included 527 independent older people. We analyzed the baseline data cross-sectionally. To measure activity behaviors, participants wore a tri-axial accelerometer for seven consecutive days. Physically active individuals were defined based on the World Health Organization recommendation guidelines on physical activity. *Sekentei* was measured using the 12-item Sekentei Scale (score range: 12–60).

**Results:**

After excluding 15 people for whom we had three or fewer days of valid accelerometer-assessed activity data, we used data from 512 participants in our analysis (average 73.4 years old; 46.9% men). Physically active individuals made up 22.3% of the sample, and the proportion of physically active men was higher than that of women. A logistic regression analysis showed that higher levels of *sekentei* were inversely associated with physical activity after adjusting for demographic factors, socioeconomic status, and health conditions (odds ratio [95% confidence interval]: 0.58 [0.36–0.91] for every 10-point increase in the Sekentei Scale score). This association was stronger in women than in men (0.66 [0.34–1.26] for men and 0.51 [0.26–1.00] for women).

**Conclusions:**

Our findings indicate that an individual’s sense of *sekentei* may be an important socio-cultural factor affecting their level of physical activity. Culturally appropriate approaches may be beneficial in addressing insufficient physical activity in older adults.

## Background

Insufficient physical activity is known to be one of the leading risk factors for non-communicable diseases, such as cardiovascular disease [1], stroke [2], diabetes [3], hypertension [4], and breast and colon cancer [5]. In addition, recent studies have reported that physical inactivity can increase the risk of dementia [6] and depression [7]. Based on these empirical findings, the member states of the World Health Organization (WHO) agreed to reduce insufficient physical activity by 10% by 2025 [8]. Based on the WHO recommendation guidelines [9], several studies have reported a gender difference in the prevalence of insufficient physical activity [10–13]. Specifically, women are more likely to engage in insufficient physical activity, particularly in Asian countries [10–13].

Determinants of physical activity are known to have several levels [14]. One factor that is fundamental in the development and maintenance of physical activity is social norms. Social norms are defined as unwritten rules about how to behave in a particular social group or culture [15]. Social norms have been incorporated into a number of theories regarding health behavior, such as the Theory of Planned Behavior [16] and Social Cognitive Theory [17]. Social norms, by definition, depend on cultural values. It is therefore important to explore the relationships between social norms and physical activity in different cultural contexts.

The Japanese concept of *sekentei* reflects the degree to which an individual feels that they must conform to social norms. The word *sekentei* consists of two words: “*seken*” and *“tei*”. “*Seken*” refers to society, community, and the public. “*Tei*” relates to reputation, honor, dignity, and appearance. *Sekentei* therefore literally means social appearance or sensitivity about one’s reputation [18]. Miyake and Yamazaki described *sekentei* as the awareness that others are observing and evaluating a person’s behaviors [19] in a way that is specific to Japanese culture [20]. It reflects Japanese behavioral principles and is commonly used to refer to an individual’s concerns about behaving in a socially acceptable manner, as judged by others [18].

Previous studies have reported that *sekentei* is associated with attitudes toward the use of care services: people with a high level of *sekentei* tend to hold attitudes that indicate that they would avoid the use of care services [21, 22]. In addition, *sekentei* has been negatively related to help-seeking intention with respect to psychological services [23]. However, there is no evidence regarding the relationship between the level of *sekentei* and health behaviors such as physical activity. *Sekentai* is a fundamental factor in determining people’s behaviors, so it is likely that it is linked with physical activity. In this study, we therefore examined the relationship between *sekentei* and objectively-measured physical activity among community-dwelling older people in Japan.

## Methods

### Study sample and data collection

We used data from the baseline survey of the Neuron to Environmental Impact across Generations Study (NEIGE Study), which was conducted in September and October 2017. Therefore, we analyzed data cross-sectionally. The NEIGE Study consisted of randomly-recruited community-dwelling independent individuals aged 65–84 years who were living in Tokamachi City, Niigata Prefecture, Japan. Tokamachi is a rural city that is located in the southernmost region of Niigata prefecture, approximately 180 km northwest of Tokyo. Tokamachi is officially registered as a heavy snowfall region during winter. As of July 1, 2017, the population size was approximately 55,000, the population density was 87.8 persons/km^2^, and the proportion of people aged 65 years and over was 36.9%.

**Fig. 1:**
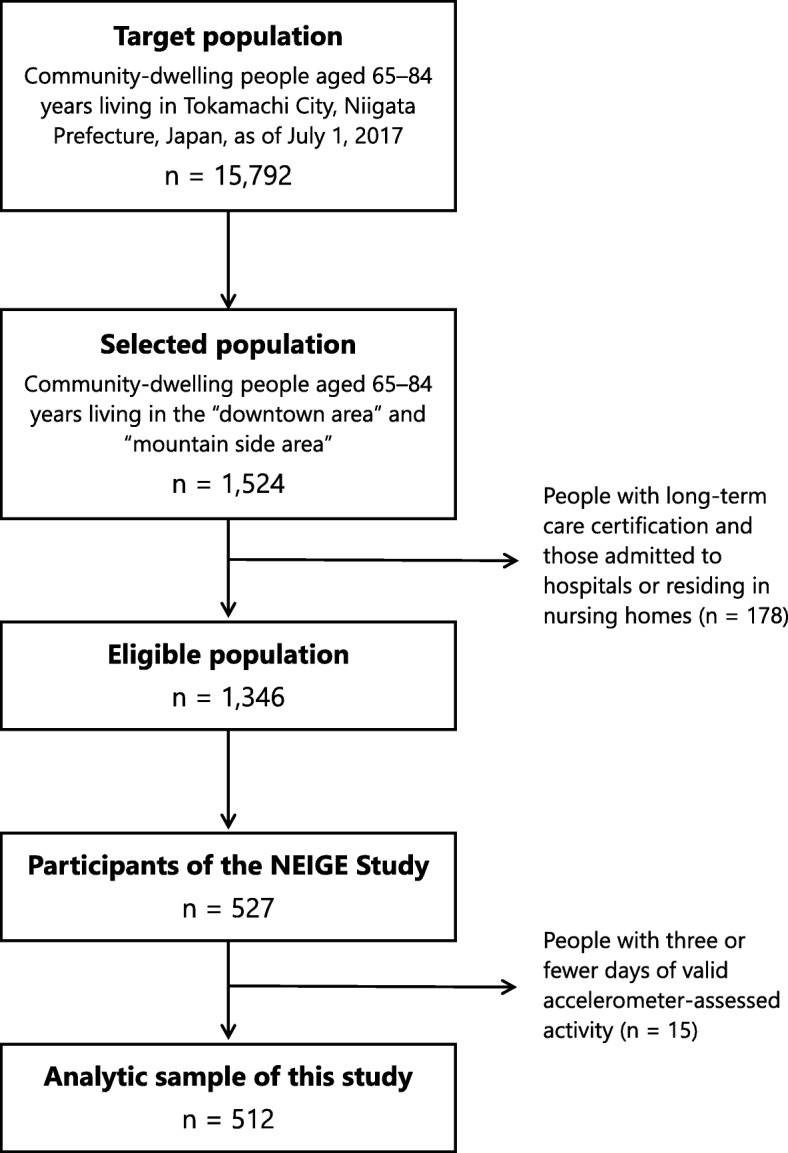
Flow diagram of study participant recruitment

To recruit the study participants, we used stratified random sampling of four groups classified by age (“65–74 years” and “75–84 years”) and area of residence within the town (“downtown area” and “mountainside area”). Among 15,792 people aged 65–84 years living in Tokamachi City (i.e., the target population; average 73.6 years old; 47.0% men), 1524 were selected. After excluding people with long-term care certification and those admitted to hospitals or residing in nursing homes, we mailed the recruitment brochure for the NEIGE Study to 1346 eligible people (average 73.9 years old; 47.0% men). In total, 527 people agreed to participate in the NEIGE Study (average 73.5 years old; 47.3% men). Figure 1 shows a flow diagram of study participant recruitment. Further information on sampling and participant demographics is given elsewhere [24].

In the NEIGE Study, we collected comprehensive information about physical, mental, cognitive, and social function via face-to-face interviews. In addition, to assess daily activity behaviors, participants were asked to wear an accelerometer.

The study protocol was reviewed and approved by the Ethical Committees of Niigata University and Tokyo Medical University. Immediately before the baseline survey, we directly informed participants of the study purpose, method, survey items, and merits of participation. All participants gave written consent to participate in this study.

### Measures

#### Physical activity

To assess activity behaviors, the participants were instructed to wear an Active style Pro HJA-750C (Omron Healthcare Co., Ltd., Kyoto, Japan) tri-axial accelerometer over the waist on an elasticated belt for seven consecutive days. They were asked to wear the belt for the entire time they were awake each day except when engaged in water-based activities (e.g., swimming and hot springs). The Active style Pro is a validated accelerometer [25–27] that provides data comparable to the devices most commonly used in studies conducted in Western countries [28, 29]. Its algorithm has been explained in detail elsewhere [25, 26]. We used the analysis application developed by Omron Healthcare Co., Ltd. to read activity data from accelerometers and the dedicated software developed by academic researchers to summarize the data.

Periods in which no acceleration signal was detected for longer than 60 consecutive minutes were defined as “non-wear”, and records indicating that the participants wore the accelerometer for at least 10 h per day were considered valid [30]. Participants with four or more valid days were included in the analysis [31]. We used 60-s data epochs and obtained estimated metabolic equivalents (METs) using analysis software. METs-based criteria were used to determine moderate-to-vigorous levels of physical activity (MVPA): ≥ 3.0 METs [32, 33].

Based on the WHO recommendation guidelines on physical activity for adults aged 65 and over [9], people who completed at least 150 min per week of MVPA in bouts of 10 min or more were regarded as physically active individuals. A 10-min MVPA session was defined as 10 or more consecutive minutes engaged in physical activity that was above the moderate intensity threshold, with an allowance for below-threshold interruptions of one or two minutes per 10 min [31].

### Sekentei

We used the Sekentei Scale [22, 34], which consists of 12 items, including “I tend to adjust my actions according to the behaviors of people around me”, “I am rather unconcerned about gossip and the way I appear to others (reverse item)”, “I avoid behavior that people laugh at”, and “I would definitely return the favor if I were cared for by or received a gift from others”. The validity and reliability of the scale have been confirmed [22]. Participants responded to 12 items on a 5-point Likert scale (“1 = strongly disagree”, “2 = disagree”, “3 = neither”, “4 = agree”, or “5 = strongly agree”). The scale scores ranged from 12 to 60, where higher scores indicate a greater sense of *sekentei*. Cronbach’s alpha in this study was 0.61.

#### Covariates

Information on age, sex, and residential area (“downtown area” or “mountainside area”) were obtained from the residential registry. The questionnaire included items measuring years of residence in the area, marital status (“married” or “not married”), current working status (“working” or “not working”), years of education (“≤ 9 years” or “≥ 10 years”), and subjective financial stability (“1 = poor”, “2 = somewhat poor”, “3 = normal”, “4 = somewhat affluent”, or “5 = affluent”). We calculated body mass index from actual measurements of height and weight (kg/m^2^), and divided the participants into three categories: “underweight” (< 18.5 kg/m^2^), “normal weight” (18.5–24.9 kg/m^2^), and “overweight” (≥ 25.0 kg/m^2^). A doctor or registered nurse conducted a medical interview to collect information on comorbidity, with a focus on the following six diagnosed diseases: cancer, hypertension, cardiovascular disease, cerebrovascular disease, dyslipidemia, and diabetes mellitus. We categorized the participants into groups based on the number of comorbid diseases: “0”, “1” and “≥ 2.”

### Statistical analyses

First, we described the characteristics of the participants. We used a t-test, chi-square test, and Mann-Whitney U-test to compare the characteristics of men and women. Second, we compared the Sekentei Scale score by the participants’ characteristics, using a t-test and one-way analysis of covariance. Statistical significance was set as *p* <  0.05 (two-tailed). Third, we examined the association between *sekentei* and the likelihood of being physically active (i.e., meeting the criteria of the WHO recommendation for physical activity), using logistic regression analysis. We used a four-step modeling strategy. Model 1 included only the Sekentei Scale score. Model 2 included age, sex, residential area, years of residence, marital status, current working status, and socioeconomic status (i.e., years of education and subjective financial stability). Model 3 used the same items as Model 2, plus health condition (i.e., body mass index and comorbidity). Previous studies have found that physical activity patterns varied significantly between men and women in a sample of the Japanese population [35]. To generate practical implications, we performed an additional stratified analysis by sex and examined whether sex could be an effect modifier. The results of the estimations are shown as odds ratios (ORs) with 95% confidence intervals (CIs). All analyses were performed using IBM SPSS 23 (IBM Corp., Armonk, NY, USA).

## Results

Among the 527 participants, 15 people had three or fewer valid days of accelerometer-assessed activity data. We therefore included 512 participants in the analysis. The 15 excluded individuals were older and the group had a higher proportion of men than the included group (76.0 years old vs. 73.4 years old; 60.0% vs. 46.9%), although these differences were not statistically significant.

Table 1 shows the participant characteristics. There were no missing values in the data. The average age was 73.4 years, and 46.9% of the participants were men. There was a greater proportion of women than men living in downtown areas. Men tended to have lived longer in the area than women, and higher proportions of men than women were married and working. With regard to socioeconomic status, 61.7% had more than 10 years of education, and 14.8% felt that they were affluent or somewhat affluent. A higher proportion of men than women had more than 10 years of education, but there was no significant sex-difference in subjective financial stability. The average score on the Sekentei Scale was 41.5, and no sex differences were observed. Finally, 22.3% were physically active individuals. The proportion of physically active men was higher than that of women (29.6% vs. 15.8%).

**Table 1:** Participant Characteristics

		Total (*n* = 512)	Men (*n* = 240; 46.9%)	Women (*n* = 272; 53.1%)	*p*-value for sex-difference
		Mean ± SD or %	Mean ± SD or %	Mean ± SD or %	
Age (years old)		73.4 ± 5.6	73.4 ± 5.6	73.4 ± 5.6	0.979^a^
Residential area	Downtown area	52.3	47.1	57.0	0.027^b^
Years of residence in the area		53.9 ± 17.5	59.1 ± 18.7	49.2 ± 15.0	< 0.001^a^
Marital status	Married	80.3	92.9	69.1	< 0.001^b^
Current working status	Working	41.6	47.9	36.0	0.007^b^
Years of education	≥ 10 years	61.7	72.9	51.8	< 0.001^b^
Subjective financial stability	Poor	4.9	3.8	5.9	0.821^c^
	Somewhat poor	18.9	20.4	17.6	
	Normal	61.3	60.4	62.1	
	Somewhat affluent	12.7	12.5	12.9	
	Affluent	2.1	2.9	1.5	
Body mass index	Underweight (< 18.5 kg/m^2^)	8.2	3.8	12.1	0.123^c^
	Normal weight (18.5–24.9 kg/m^2^)	73.8	78.8	69.5	
	Overweight (≥ 25.0 kg/m^2^)	18.0	17.5	18.4	
Comorbidity	0	34.4	32.9	35.7	0.070^c^
	1	36.5	32.5	40.1	
	≥ 2	29.1	34.6	24.3	
Sekentei Scale score (ranging from 12 to 60)		41.5 ± 5.0	41.4 ± 4.8	41.6 ± 5.1	0.767^a^
Physically active		22.3	29.6	15.8	< 0.001^b^

*SD* standard deviation

^a^t-test, ^b^chi-square test, ^c^ Mann-Whitney U-test

In Table 2, we compared the Sekentei Scale scores with participant characteristics. The older group (people aged 75–84 years) had higher levels of *sekentei* than the younger group (people aged 65–74 years). People who had lived in the area for 54 years or more (above the median) and who were not employed had a higher Sekentei Scale score. The Sekentei Scale score was lower among physically active individuals than physically inactive individuals.

**Table 2:** Sekentei Scale score by participant characteristics

		Sekentei Scale score (ranging from 12 to 60)	*p*-value
		Mean ± SD	
Age	65–74 years old	40.9 ± 5.0	0.001^a^
	75–84 years old	42.4 ± 4.8	
Residential area	Downtown area	41.8 ± 5.0	0.185^a^
	Mountain side area	41.2 ± 5.0	
Years of residence in the area	≥ 54 years (above median)	42.0 ± 4.8	0.018^a^
	≤ 53 years (below median)	41.0 ± 5.1	
Marital status	Married	41.5 ± 4.8	0.674^a^
	Unmarried	41.7 ± 5.6	
Current working status	Working	40.9 ± 5.2	0.028^a^
	Not working	41.9 ± 4.8	
Years of education	≥ 10 years	41.5 ± 5.0	0.771^a^
	≤ 9 years	41.4 ± 5.0	
Subjective financial stability	Poor	39.3 ± 6.1	0.165^b^
	Somewhat poor	41.1 ± 5.0	
	Normal	41.7 ± 4.7	
	Somewhat affluent	41.8 ± 5.4	
	Affluent	42.5 ± 6.0	
Body mass index	Underweight (< 18.5 kg/m^2^)	40.3 ± 4.1	0.104^b^
	Normal weight (18.5–24.9 kg/m^2^)	41.8 ± 4.9	
	Overweight (≥ 25.0 kg/m^2^)	41.0 ± 5.5	
Comorbidity	0	41.1 ± 4.9	0.320^b^
	1	41.7 ± 5.1	
	≥ 2	41.8 ± 4.9	
Physically active	Yes	40.4 ± 5.4	0.008^a^
	No	41.8 ± 4.8	

*SD* standard deviation

^a^t-test, ^b^one-way analysis of covariance

Table 3 illustrates the association between *sekentei* and physical activity in the total sample. In Model 1 (unadjusted model), the Sekentei Scale score was inversely associated with physical activity, such that a higher score was associated with less physical activity (OR [95% CI]: 0.57 [0.38–0.87] for every 10-point increase in the score). This association persisted after adjusting for demographic factors, socioeconomic status, and health conditions in Models 2 and 3 (0.60 [0.38–0.94] in Model 2, and 0.58 [0.36–0.91] in Model 3).

**Table 3:** The association between *sekentei* and physical activity in the total sample

		Model 1		Model 2		Model 3	
		OR	(95% CI)	OR	(95% CI)	OR	(95% CI)
Sekentei Scale score (every 10-point increase)		0.57	(0.38–0.87)	0.60	(0.38–0.94)	0.58	(0.36–0.91)
Age (every 1-year increase)				0.89	(0.84–0.93)	0.88	(0.84–0.93)
Sex (ref. women)	Men			2.35	(1.43–3.84)	2.24	(1.35–3.73)
Residential area (ref.: mountain side area)	Downtown area			0.97	(0.61–1.53)	0.91	(0.57–1.45)
Years of residence in the area (every 10-year increase)				1.07	(0.93–1.23)	1.05	(0.91–1.21)
Marital status (ref.: unmarried)	Married			0.90	(0.47–1.73)	0.84	(0.43–1.63)
Current working status (ref.: not working)	Working			1.01	(0.64–1.59)	0.98	(0.62–1.56)
Years of education (ref.: ≤ 9 years)	≥ 10 years			1.25	(0.75–2.10)	0.77	(0.46–1.31)
Financial stability				1.42	(1.06–1.92)	1.41	(1.05–1.91)
Body mass index (ref.: normal weight)	Underweight					0.73	(0.29–1.83)
	Overweight					0.61	(0.32–1.17)
Comorbidity (ref.: none)	1					0.56	(0.32–0.97)
	≥ 2					1.20	(0.69–2.08)

*CI* confidence interval, *OR* odds ratio

Table 4 indicates the results of the sex-stratified analyses. After adjusting for demographic factors, socioeconomic status, and health conditions, a higher Sekentei Scale score was significantly associated with less physical activity in women, but not in men (0.66 [0.34–1.26] for men and 0.51 [0.26–1.00] for women).

**Table 4:** The association between *sekentei* and physical activity by sex (based on Model 3)

		Men		Women	
		OR	(95% CI)	OR	(95% CI)
Sekentei Scale score (every 10-point increase)		0.66	(0.34–1.26)	0.51	(0.26–1.00)
Age (every 1-year increase)		0.88	(0.82–0.94)	0.89	(0.82–0.97)
Residential area (ref.: mountain side area)	Downtown area	0.91	(0.49–1.70)	0.90	(0.43–1.91)
Years of residence in the area (every 10-year increase)		1.05	(0.88–1.25)	1.08	(0.83–1.41)
Marital status (ref.: unmarried)	Married	0.41	(0.13–1.26)	1.14	(0.47–2.78)
Current working status (ref.: not working)	Working	0.62	(0.34–1.15)	1.79	(0.87–3.71)
Years of education (ref.: ≤ 9 years)	≥ 10 years	0.57	(0.27–1.18)	0.97	(0.45–2.11)
Financial stability		1.46	(0.97–2.17)	1.43	(0.89–2.29)
Body mass index (ref.: normal weight)	Underweight	0.86	(0.15–4.74)	0.60	(0.20–1.83)
	Overweight	0.95	(0.42–2.18)	0.28	(0.08–0.99)
Comorbidity (ref.: none)	1	0.56	(0.26–1.21)	0.56	(0.25–1.27)
	≥ 2	1.35	(0.65–2.80)	1.09	(0.43–2.76)

*CI* confidence interval, *OR* odds ratio

As a sensitivity analysis, we divided the Sekentei Scale data into quartiles, instead of using the score as a continuous value. The trends in the associations in both the total sample and the sex-stratified samples were not necessarily linear or statistically significant, but were consistent with those shown in Tables 3 and 4. Higher scores for the Sekentei Scale were correlated with less likelihood of being physically active, and this association was stronger in women (see Additional file 1: Table S1).

## Discussion

In this study, we used baseline data from the NEIGE study of randomly-sampled community-dwelling older Japanese people, and examined the relationship between *sekentei* and physical activity, objectively measured using a tri-axial accelerometer. We found that higher *sekentei* levels were associated with lower physical activity. We hypothesized that *sekentei* consciousness is a behavioral principal in Japanese culture, so may be an important factor in addressing insufficient physical activity in Japanese society. However, to our knowledge, no previous studies have examined the relationship between *sekentei* and health behaviors, including physical activity. These findings contribute to our understanding of the social determinants of physical activity.

One plausible mechanism underlying the association between higher *sekentei* levels and lower physical activity could be related to ageism. Ageism is stereotyping, prejudice, and discrimination against people on the basis of their age [36]. Negative stereotyped images of older people can include “sick”, “impotent”, “ugly”, “senile”, “mentally ill”, “useless”, “isolated”, “poor”, and “depressed” [37]. People with high levels of *sekentei* are more likely to behave in ways that others will approve of or consider appropriate in society [38], so they may tend to refrain from living active daily lives. This might result in less physical activity among people with high *sekentei* levels. North and Fiske carried out a cross-cultural meta-analysis and found negative attitudes toward older people (i.e., ageism) were higher in Asian countries than Western countries, and were greatest in East Asia, including Japan [39]. The relationship between *sekentei* and physical activity, which can be influenced by the level of ageism, might therefore be particularly strong in Japan.

The inverse association between *sekentei* and physical activity was stronger in women than in men. Previous studies reported a gender gap in insufficient physical activity in several regions in the world, including high-income Pacific Asian countries. Specifically, the proportion of women who engaged in insufficient physical activity was higher than that of men [10–13], especially with respect to leisure time [13]. Older women tended to spend more time in the community than men [40], because most women in the study generation had been full-time housewives. They might therefore have been more sensitive to the acceptance of the community, and thus engaged in less physical activity. Moreover, older people in Japan have been found to have stronger gender-role attitudes than younger people [41], such that many of them (including both men and women) agree that housework is the primary responsibility of women. Such norms regarding gender roles might affect the association between *sekentei* and physical activity. That is, women with higher *sekentei* levels might be more likely to stay at home and engage in housekeeping work, and thus be less physically active (i.e., adherence to the WHO recommendation guidelines). Indeed, fewer of the physically active individuals in this study were women than men (see Table 1). A previous study pointed out that cultural norms and traditional roles in society might lead to reduced participation in physical activity among women [10]. In Japan, the norms that lead to reduced physical activity in women might be related to *sekentei*.

Our data indicate that physical activity depends not only on individual factors (e.g., age, sex, socioeconomic status) but also on the interaction between the individual and the society to which they belong. *Sekentei* is a psychosocial characteristic that is unique to Japanese people and forms over a long period of time throughout one’s life [20]. It is reported that *sekentei* levels among Japanese people are generally lower than ever before [20]. This is because of recent increases in urbanization and reductions in disparity between urban and rural areas, particularly with regard to information and transportation [20]. However, it may not be easy to adjust one’s sense of *sekentei*. Assessments of *sekentei* may be useful in promoting physical activity among older people. For example, when supporting behavioral changes in physical activity, health professionals and health educators could assess the *sekentei* level of an individual and use that information to suggest a feasible form of physical activity that they could easily incorporate in daily life. For policymakers, developing strategies based on an understanding of *sekentei* levels in the target community might be helpful in decreasing insufficient physical activity among local residents. Culturally appropriate approaches are likely to be important in encouraging people to participate in physical activity.

Although unique to Japanese culture, the concept of *sekentei* has core elements that are common across Asian cultures [18]. Our findings can therefore likely be applied to other Asian nations. A previous report showed that the proportion of individuals who engaged in insufficient physical activity was higher in high-income Asian Pacific countries (e.g., South Korea and Singapore) compared with the global average [10]. Our findings might contribute to policies aimed at decreasing the proportion of individuals who engage in insufficient physical activity in Asian nations that have similar characteristics to Japan.

There are several limitations to the present study. First, the sample may have contained especially healthy individuals. Our study participants were randomly-sampled, but they tended to be healthy and wealthy compared with the Japanese average, and perhaps physically inactive people were less likely to participate. There might therefore have been a selection bias. Second, the internal consistency of the Sekentei Scale in the current study was not high (Cronbach’s alpha = 0.61). The scale was developed using data from young, middle-aged, and older people [22, 34]. However, this study was limited to those aged 65–84 years, which might led to the low internal consistency. Third, although accelerometers provide objective measures, they cannot discern activity for a particular purpose. It could therefore be helpful to evaluate domain-specific activity measures as well as accelerometer-based measures. Fourth, this was a cross-sectional study. A longitudinal study would be necessary to confirm causal relationships. Finally, because the target community was limited to one geographical region, care should be taken in generalizing the findings.

## Conclusions

This study revealed that a higher sense of *sekentei* was inversely associated with physical activity, objectively measured via a tri-axial accelerometer, among community-dwelling older Japanese people. This association was greater in women than in men. The socio-cultural background of local residents, including specific factors such as sense of *sekentei*, may be useful to consider when developing more effective ways to resolve insufficient physical activity and promote healthy aging.

## Supplementary information

**Additional file 1: Table S1.** The association between *sekentei* and physical activity, using Sekentei Scale scores grouped by quartile.

## Data Availability

The datasets used and analyzed during the current study are available from the corresponding author on reasonable request.

## Abbreviations

CI: Confidence interval
METs: Metabolic equivalents
MVPA: Moderate-to-vigorous physical activity
NEIGE Study: the Neuron to Environmental Impact across Generations Study
OR: Odds ratio
WHO: World Health Organization
