# Supplementary material for: Sekentei and objectively-measured physical activity among older Japanese people: a cross-sectional analysis from the NEIGE study
Source: BMC Public Health. 2019 Oct 22;19:1331. doi: 10.1186/s12889-019-7702-4 (PMC6805600; doi:10.1186/s12889-019-7702-4)
Supplement: Supplementary file 1 — Additional file 1: Table S1. The association between sekentei and physical activity, using Sekentei Scale scores grouped by quartile. [file 12889_2019_7702_MOESM1_ESM.docx]

| **Additional file 1: Table S1.** The association between *sekentei* and physical activity, using Sekentei Scale scores grouped by quartile | | | | | | | | | |
| --- | --- | --- | --- | --- | --- | --- | --- | --- | --- |
|  |  | **Total sample**^a^ | |  | **Men**^b^ | |  | **Women**^b^ | |
|  |  | OR | (95% CI) |  | OR | (95% CI) |  | OR | (95% CI) |
| Sekentei Scale score | Q1 (highest) | 0.61 | (0.32–1.14) |  | 0.75 | (0.33–1.74) |  | 0.42 | (0.15–1.20) |
|  | Q2 | 0.47 | (0.25–0.90) |  | 0.51 | (0.21–1.21) |  | 0.43 | (0.15–1.18) |
|  | Q3 | 0.61 | (0.33–1.14) |  | 0.63 | (0.27–1.45) |  | 0.65 | (0.26–1.64) |
|  | Q4 (lowest) | 1.00 | Reference |  | 1.00 | Reference |  | 1.00 | Reference |
| CI: confidence interval. OR: odds ratio. | | | | | | | | | |
| a: Adjusting for age, sex, residential area, years of residence in the area, marital status, current working status, years of education, financial stability, body mass index, and comorbidity. | | | | | | | | | |
| b: Adjusting for age, residential area, years of residence in the area, marital status, current working status, years of education, financial stability, body mass index, and comorbidity. | | | | | | | | | |
